# Neurodevelopmental Trajectories and Psychiatric Morbidity: Lessons Learned From the 22q11.2 Deletion Syndrome

**DOI:** 10.1007/s11920-021-01225-z

**Published:** 2021-02-24

**Authors:** Ania M. Fiksinski, Maude Schneider, Janneke Zinkstok, Danielle Baribeau, Samuel J. R. A. Chawner, Jacob A. S. Vorstman

**Affiliations:** 1 Department of Psychiatry, Brain Center, University Medical Center Utrecht, Utrecht, The Netherlands; 2 Dalglish Family 22q Clinic for Adults with 22q11.2 Deletion Syndrome, Toronto General Hospital, University Health Network, Toronto, Canada; 3 Clinical Genetics Research Program, Centre for Addiction and Mental Health, Toronto, Ontario Canada; 4 Clinical Psychology Unit for Intellectual and Developmental Disabilities, Faculty of Psychology and Educational Sciences, University of Geneva, Geneva, Switzerland; 5 Department of Neurosciences, Center for Contextual Psychiatry, KU Leuven, Leuven, Belgium; 6 Department of Psychiatry, Hospital for Sick Children, Toronto, ON Canada; 7 Department of Psychiatry, University of Toronto, Toronto, ON Canada; 8 Cardiff University Centre for Human Developmental Science, School of Psychology, Cardiff University, Cardiff, UK; 9 MRC Centre for Neuropsychiatric Genetics and Genomics, School of Medicine, Cardiff University, Cardiff, UK; 10 The Centre for Applied Genomics, Program in Genetics and Genome Biology, The Hospital for Sick Children, Toronto, ON Canada

**Keywords:** 22q11.2 deletion, Neurodevelopment, Psychiatry, Variable penetrance, Copy number variant, Genetic model

## Abstract

**Purpose of Review:**

The 22q11.2 deletion syndrome (22q11DS) is associated with a broad spectrum of neurodevelopmental phenotypes and is the strongest known single genetic risk factor for schizophrenia. Compared to other rare structural pathogenic genetic variants, 22q11DS is relatively common and one of the most extensively studied. This review provides a state-of-the-art overview of current insights regarding associated neurodevelopmental phenotypes and potential implications for 22q11DS and beyond.

**Recent Findings:**

We will first discuss recent findings with respect to neurodevelopmental phenotypic expression associated with 22q11DS, including psychotic disorders, intellectual functioning, autism spectrum disorders, as well as their interactions. Second, we will address considerations that are important in interpreting these data and propose potential implications for both the clinical care for and the empirical study of individuals with 22q11DS. Third, we will highlight variable penetrance and pleiotropy with respect to neurodevelopmental phenotypes in 22q11DS. We will discuss how these phenomena are consistently observed in the context of virtually all rare pathogenic variants and that they pose substantial challenges from both a clinical and a research perspective.

**Summary:**

We outline how 22q11DS could be viewed as a genetic model for studying neurodevelopmental phenotypes. In addition, we propose that 22q11DS research can help elucidate mechanisms underlying variable expression and pleiotropy of neurodevelopmental phenotypes, insights that are likely relevant for 22q11DS and beyond, including for individuals with other rare pathogenic genetic variants and for individuals with idiopathic neurodevelopmental conditions.

## Introduction

22q11.2 deletion syndrome (22q11DS) is caused by a heterozygous microdeletion of region 11.2 on the long arm of chromosome 22 [1]. The prevalence of 22q11DS is currently estimated to range from 1 per 3000 to 1 per 6000 live births, with approximately 90–95% of cases not inherited from parents, i.e., arising from a de novo event [2]. 22q11DS is characterized by a complex and variable phenotype, including somatic, cognitive, and psychiatric manifestations. Shortly, after the discovery of the genetic etiology of this syndrome in the 1990s [1], studies emerged reporting an increased rate of schizophrenia and related psychotic disorders among individuals with 22q11DS [3–6]. These observations prompted several investigators across different sites to examine cognitive and behavioral characteristics of individuals from early childhood to adulthood. Taken together, these studies identified increased rates of a wide range of neurodevelopmental and psychiatric symptoms in this population beginning in early childhood [7•].

Starting over a decade ago, international collaborations such as the Psychiatric Genomic Consortium [8] began pooling case-control cohorts into large samples for genome-wide association studies for a range of psychiatric phenotypes. This approach demonstrated how large-scale international collaborations can successfully achieve certain scientific analyses that remain out of reach for independently working research groups. The International 22q11DS Brain Behavior Consortium (IBBC), funded by the US National Institute of Mental Health (NIMH), followed this example by bringing together 22 different sites across the world [9]. The aims of the IBBC included the identification of genetic mechanisms modifying the variable expression of schizophrenia in this syndrome, as well as further characterization of other behavioral and cognitive consequences of the syndrome. Following the same rationale and also funded by the NIMH, more recently, the Genes To Mental Health (G2MH) Network was created (www.genes2mentalhealth.com), joining the efforts of numerous international researchers to examine these same questions in individuals with rare genomic variants associated with neurodevelopmental and psychiatric disorders (including but not limited to 22q11DS).

Here, we will provide an overview of the current understanding of both psychiatric and cognitive manifestations observed in individuals with 22q11DS and what is known about the relationship between both domains. We will discuss potential implications for studies of and clinical care for individuals with 22q11DS and other rare variants, as well as highlight implications for idiopathic psychiatric disorders.

## Psychiatric Observations Across the Lifespan and Associated Considerations

A landmark study by the IBBC [10] combined psychiatric data from multiple international sites on 1401 individuals with 22q11DS aged 6–69 and resulted in the largest study of psychiatric morbidity across the lifespan in 22q11DS [10]. ADHD (predominantly inattentive type rather than hyperactivity) is particularly common (in 37%) during childhood (6–12 years), with prevalence decreasing to 15% in adulthood. Autism spectrum disorders (ASD) are diagnosed in approximately 10–40% of individuals with 22q11DS. Anxiety disorders are frequent across the lifespan (in 31%), but specific behavioral manifestations and symptoms of anxiety tend to evolve with age, with specific phobia, social phobia, and separation anxiety disorder being more prevalent in younger age groups and panic disorder more frequent among adults. Mood disorders are relatively uncommon in children, but their prevalence, especially of major depressive disorder, increases with age and reaches 20% in older adults (≥ 36 years). Bipolar mood disorder, conduct disorder, and substance use disorders do not appear to be increased in this population [10, 11]. Few studies have studied comorbidity, but those that have report frequent cooccurrence of two or more psychiatric disorders, particularly between ADHD and anxiety disorders, and between anxiety, mood, and schizophrenia [10, 12, 13].

22q11DS is recognized as the highest known single genetic risk factor for schizophrenia, with approximately 20–25% of individuals developing this severe psychiatric illness by adulthood [4]. 22q11DS offers a unique opportunity to understand the neurodevelopmental pathophysiology of schizophrenia, including prospectively during early developmental stages [14]. Efforts have recently been devoted to characterizing early prodromal development of psychosis in 22q11DS, in line with the clinical high-risk (CHR) model of psychosis (for a review, see [15]). In a recent collaborative study which assessed 692 non-psychotic individuals with 22q11DS aged 6–55 years with the Structured Interview for Psychosis Risk Syndromes (SIPS [16]), 54% of the sample was diagnosed with at least one psychotic symptoms (positive, negative, or disorganized) of moderate to high severity [17]. Importantly, clinical manifestations of schizophrenia in 22q11DS largely converge with that for idiopathic schizophrenia [18–20]. However, some subtle differences have been reported; compared to other populations at risk for psychosis, individuals with 22q11DS are characterized by more severe negative symptoms of schizophrenia, including social anhedonia and avolition [21–23]. This suggests that 22q11DS might represent a good model to study negative symptoms that represent a yet unmet need in the management of schizophrenia [24, 25]. The presence of CHR criteria (e.g., attenuated positive symptoms of psychosis above a certain frequency and duration threshold) has been shown to predict conversion to psychosis within 36 months in 27% of 22q11DS cases [26], which is comparable to idiopathic schizophrenia [27]. However, the fact that 4.5% of individuals with 22q11DS converted to psychosis *without* meeting CHR criteria points to the need to identifying predictors beyond classical CHR criteria for psychotic disorders in this population [26].

The prevalence rates of psychiatric manifestations in 22q11DS should be interpreted in light of ascertainment strategies, which vary across studies. Particularly those sites that recruit within a clinical setting may be more likely to include individuals who already have psychiatric symptoms. Further, the majority of studies include only individuals already known to have a diagnosis of 22q11DS and will therefore not capture asymptomatic individuals who have not been referred for genetic testing. One of the few population-based epidemiological studies of 22q11DS used medical and genomic data from a Danish nationwide registry, thereby capturing individuals in the population with 22q11DS who had not received a 22q11DS diagnosis within a clinical setting. Here, a similar pattern of psychiatric disorders was reported, but with lower prevalence rates (e.g., 7% for psychosis) compared to previous findings [28]. However, prevalence estimates in this study were derived from medical records, potentially underestimating the true prevalence of psychiatric conditions in the 22q11DS population. Also, medical record validity may be affected by diagnostic overshadowing and differences in regional diagnostic expertise and practices. Prevalence rates in studies may also be influenced by the assessment methods used to query psychopathology. For example, differences in the reported rates of ASD between studies are likely partly explained by variability in the use of appropriate assessment methods for this condition [29]. In addition, in the general population the comorbidity between ADHD and ASD symptoms is well-established [30–32] and may therefore also account for some of the variation in the reported rates of both disorders in 22q11DS across studies. Lastly, studies of 22q11DS have highlighted issues with current binary, categorical conceptualization of psychopathology, whereby disorders are either present or absent. The clinical reality for many individuals with 22q11DS is such that even in the absence of meeting criteria for a formal psychiatric diagnosis, clinically relevant psychiatric symptoms are present [33, 34].

The heterogeneity of cognitive, neurodevelopmental, and psychiatric symptoms in 22q11DS mirrors the high degree of heterogeneity regarding *physical* manifestations, which can include wide-ranging combinations of brain, palate, cardiac, and/or immune effects. Indeed, the observed phenotypic variability in 22q11DS is consistent with the phenomena of pleiotropy, incomplete penetrance, and variable expressivity (see Glossary), reported consistently in many rare CNVs [35, 36]. Studies of psychiatric prevalence in 22q11DS raise important issues for the field of psychiatry. First, findings demonstrate that the risk conferred by one genetic variant is not necessarily specific. Indeed, 22q11DS is highly pleiotropic, conferring risk for several psychiatric conditions across the lifespan that may or may not manifest independently of one another. Second, observations highlight that psychiatric phenotypes are not fully penetrant. For example, not every individual with 22q11DS will develop schizophrenia; indeed, the majority will not. Third, the findings collectively raise issues surrounding current psychiatric classification, in that the range and comorbidity of psychiatric phenotypic expressions associated with a specific genetic variant is oftentimes difficult to capture by the categorical classification. Many individuals with 22q11DS who do not meet all criteria for a specific psychiatric disorder may nevertheless experience clinically distressing symptoms in that domain, highlighting the need for a more dimensional approach [37, 38].

## 22q11.2 Deletion Syndrome as a Model for Studying Neuropsychiatric Conditions

22q11DS has been highlighted as a valuable model for studying neuropsychiatric conditions, as it is one of the strongest known risk factors for development of psychiatric illness. Increasing identification of 22q11DS prenatally or very early in life [1] permits in-depth prospective study of clinical and/or environmental longitudinal predictors, whilst minimizing ascertainment bias. As a high-risk group, it provides a magnifying lens to study how modifying factors such as additional genetic variability (including deletion size, breakpoints, structure of the intact chromosome, and other rare-rare or rare-common variant interactions) may contribute to highly variable clinical presentations. While results of such studies may be specific to 22q11DS, findings have implications beyond this specific disorder. In particular, 22q11DS can serve as a helpful example to understand the molecular mechanisms involved in the emergence of neuropsychiatric conditions; as a model to understand the early trajectories of neuropsychiatric disorders, in particular schizophrenia; and as a way elucidate mechanism that influence variable expression and pleiotropy in individuals with high-impact pathogenic genetic variants.

**Fig. 1:**
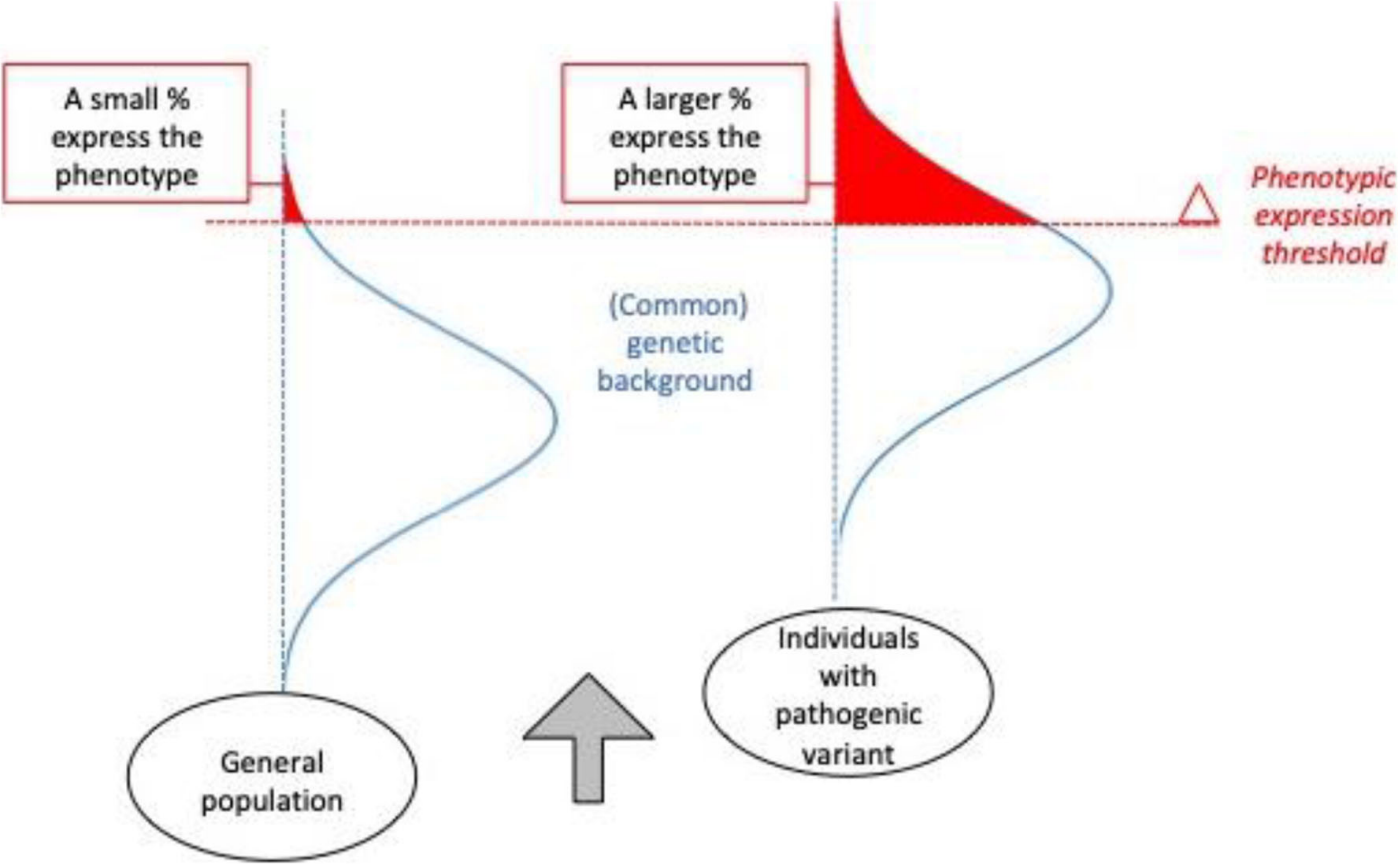
High-impact genetic variants exert their impact on neuropsychiatric phenotypes in the context of common genetic variation. In the general population (left), the cumulative burden of common genetic variants that impact neurodevelopment are distributed (blue) such that only a very small proportion of individuals will be above the phenotype expression threshold (red) and develop a neurodevelopmental disorder. In individuals with a high-impact variant (right), such as the 22q11.2 deletion, the same genetic background factors influence risk for neuropsychiatric outcomes. However, due to the elevated baseline risk conferred by the variant, a much larger proportion will express the phenotype

Regarding genetic modifiers, so far, data do not support a 1:1 relationship between any individual gene in the 22q11.2 region and a particular phenotype [39]. Deletion size and breakpoint location vary for a minority of subjects [40–42] and may be associated with modest variability in clinical expression [43], or brain structure [44]. Recently, research efforts have shifted towards studying two-hit or multihit hypotheses to explain variable penetrance in 22q11DS. Emerging data suggest that common and rare variants in the intact (non-deleted) chromosome may affect proclivity towards psychosis [45], or Parkinson’s disease in some families with 22q11DS [46]. For example, other rare CNVs elsewhere in the genome may confer additional risk for adverse outcomes in 22q11DS [47, 48]. Broadly conceptualized, results may support a model of neuropsychiatric disorder, whereby in the general population, one’s proclivity towards (or resilience against) specific manifestations (e.g., cognitive impairment, psychiatric symptoms) may be partly determined by one’s (mostly inherited) profile of common genetic variants [49]. The vast majority of people in the general population do not express the more severe developmental or psychiatric phenotypes such as schizophrenia, ASD, or intellectual disability. However, within the general population, there are subsets of people who are affected by a rare, pathogenic variant, such as the 22q11.2 deletion. Over the past two decades, many studies have shown that in these individuals, the prevalence of severe phenotypes is increased compared to the general population, with variation in penetrance dependant on the specific genetic variant (e.g., [50]). In any *individual* carrier of a specific rare pathogenic variant associated with developmental and psychiatric phenotypes, the expression of a specific phenotype is likely under the influence of the (mostly inherited) profile of common genetic variants [51•] (Fig. 1).

Within 22q11DS, the investigation of additional genetic modifiers has largely focused on the heterogeneity in psychosis outcomes. A proof-of-principle study using whole-genome sequencing (WGS) in a small but well-phenotyped sample of individuals with 22q11DS reported higher burden of additional rare mutations in protein-coding neurofunctional genes in individuals with 22q11DS diagnosed with schizophrenia, compared to those without schizophrenia [52]. Also, preliminary findings indicate that individuals with 22q11DS and schizophrenia carry an excess of rare deletions that overlap with protein-coding genes when contrasted to 22q11DS individuals without schizophrenia [47]. One recent IBBC study included 519 unrelated patients and focused on genomic mechanisms that contribute to schizophrenia risk using WGS and microarray data. [53] The results showed that the same common genetic variation (summarized by the polygenic risk score (PRS) for schizophrenia) associated with schizophrenia in the general population, also modify schizophrenia expression in the context of the 22q11.2 deletion. Specifically, increased PRS in the subset of individuals with 22q11DS and schizophrenia was identified compared to those without schizophrenia [53]. At present, PRS is not suitable for individual or clinical prediction of psychosis risk [54], but its potential is being widely investigated, including whether it may be used in multimodal prediction tools to help identify those at risk and tailor preventive and/or early intervention strategies to those at increased risk of developing psychosis [55•]. Indeed, a recent study by the IBBC applied PRS for schizophrenia and PRS for intellectual function, both derived from studies in the general population, in a cohort of individuals with 22q11DS. The results showed that risk stratification is possible and yields results that are getting closer to those required for clinical use [56•].

The heterogeneity of the 22q11DS phenotype may be partly explained by the presence of regulatory genes in the 22q11.2 locus [57]. These regulatory genes may have variable impacts on gene expression outside the deleted region. For example, the 22q11.2 region contains several miRNAs [57]. An miRNA is a small, non-coding RNA molecule that regulates gene expression [58]. In addition, the DiGeorge Syndrome Critical Region Gene 8 (*DGCR8*), a gene located in the commonly deleted region, encodes a key miRNA processing protein [57]. Haploinsufficiency of the *DGCR8* gene has been shown to disrupt miRNA functioning [57, 59, 60], and this could directly or indirectly affect the expression of neuropsychiatric risk genes elsewhere in the genome through altered up- or downregulated gene expression [59, 60]. This hypothesis is supported by recent data from mouse studies suggesting that haploinsufficiency of DGCR8 causes downregulation of the dopamine D_2_ receptor in relevant brain regions for schizophrenia [59, 60], elevated DRD1 expression [61], and age-dependent ventricular enlargement [61]. These data may help explain etiological mechanisms underlying schizophrenia, especially the onset of psychotic symptoms in adolescence.

Environmental factors that are known to influence risk of psychiatric illness in the general population, e.g., trauma and stress, have not yet been extensively investigated in 22q11DS [62, 63]. Large-scale prospective studies following well-phenotyped 22q11DS individuals that include environmental influences may provide information on the various mechanisms that play a role in brain-related phenotypes seen in 22q11DS.

Overall, 22q11DS presents an opportunity to study genetic and molecular mechanisms, as well as other clinical and environmental predictors and modifiers of neuropsychiatric expression over the lifespan, with potential implications for all neuropsychiatric conditions. Prospective, longitudinal studies of 22q11DS have helped identify markers for psychosis that precede onset of the illness, laying the foundations for development of preventive measures or early intervention [9, 64]. A body of research (reviewed here [65]) has identified several cognitive and psychiatric factors in childhood that are associated with subsequent development of psychosis. Prenatal and neonatal factors may affect its longitudinal course to some extent [66, 67], and a recent study demonstrated a significant association between parental anxiety and depression with psychopathology in offspring with 22q11DS [68].

Findings from studies of 22q11DS may help guide patients and families and support optimal clinical care. However, it is becoming increasingly evident that they may also have broader implications: 22q11DS, as a high-risk group, provides a unique lens for identifying genetic, clinical, and environmental factors that inform on the developmental trajectories of psychiatric conditions. Research in this field has potential for leading the way in identifying the mechanisms that contribute to the development of psychiatric disorders and for developing early interventions to prevent their onset.

## Cognitive Functioning and Trajectories in 22q11DS

A core feature of 22q11DS is intellectual disability (i.e., significant impairments in cognitive and daily life skills, and IQ level below 70) with approximately 45% of patients falling in this category [1, 69]. However, this statistic of penetrance relies on a categorical view, thereby failing to capture the high variability of expression of the cognitive phenotype, both between and within individuals. Decades of longitudinal studies of individuals with 22q11DS across all age groups have resulted in several insights with relevant ramifications to affected individuals and their potential implications for studying populations with other pathogenic variants [65, 70].

In young children with 22q11DS, delayed milestones, both globally or, more specifically regarding language, are frequently observed [71]. Once standardized cognitive testing is feasible, intellectual functioning is most commonly evaluated within the range of mild intellectual disability (i.e., full scale IQ (FSIQ) 55–70) to borderline intellectual functioning (FSIQ between 70 and 85). A minority of patients have scores in the more extreme ends of the distribution, including around the minimum measurable IQ score (e.g., IQ = 40), as well as IQ scores around 120 [72, 73]. On average, the deletion causes a negative shift of the IQ distribution, of which the mean is 100 in the general population with a standard deviation of 15 [74]. Hence, in 22q11DS, the IQ distribution has shifted ~2 SD to the left, while range and variance of the distribution otherwise are not altered in comparison to the IQ distribution in the general population [43, 75].

In the general population, the effect of parental level of cognitive functioning on offspring cognitive functioning, also reflected in the role of common (shared) genetic variance for IQ, has been well established [76]. Evidence from studies of 22q11DS [77, 78] and other pathogenic variants (e.g., [79]) in support of a similar mechanism is emerging. A recent study reported that while IQ scores were ~ 30 points lower in 22q11DS patients compared to their unaffected parents, the distribution was significantly associated to the parental distribution [80]. This is in line with the finding that common genetic variants associated with IQ, or proxies thereof, in the general population (IQ polygenic score [81]) are similarly important in shaping the cognitive phenotype in the context of the high-impact 22q11.2 deletion [56].

While severe levels of intellectual disability are uncommon in children, they are more frequently observed in adults with 22q11DS [71, 73, 82] suggesting that cognitive abilities may not be stable in all individuals with 22q11DS [83]. Several studies, including one which studied the cognitive trajectories of 718 individuals with 22q11DS, have reported that, overall, individuals with 22q11DS show a modest but significant decline in IQ throughout development [69, 75, 83]. These longitudinal IQ-data suggest that in childhood and adolescence, the observed typical decline in 22q11DS mostly reflects a slower pace in cognitive development compared to typically developing peers [84]. In adulthood, however, the observed decline suggests that individuals with 22q11DS are losing cognitive capacities at a faster pace compared to the general population [74].

A subgroup of individuals with 22q11DS shows a decline in IQ in excess of what is expected even *within* this population. This IQ-decline, most prominent in Verbal IQ, is associated with a further elevated risk (compared to the baseline risk of ~25% [1]) of developing schizophrenia [69]. Such findings are consistent with longitudinal studies in the general population, which report that individuals who are at high risk or later developed a psychotic disorder showed increasing cognitive impairments over time, especially during adolescence [85–89]. There is emerging evidence that cognitive decline may in fact not only be a phenotypic precursor of full-blown schizophrenia, but may represent an earlier stage of the biological disease process and share part of the same genetic etiology. For example, a recent study of 540 idiopathic schizophrenia patients reported that those individuals with a significant cognitive decline had the highest schizophrenia polygenic risk score (SCZ-PRS), as compared to the individuals who remained cognitively stable and/or were already more severely cognitively impaired from an early age onwards [90]. A recent study from the 22q11DS IBBC reported findings in accordance with this. In this sample of 962 individuals with 22q11DS, SCZ-PRS was significantly elevated in individuals with cognitive decline compared to those without, indicating that common genetic risk factors for schizophrenia contribute to cognitive decline. At the same time, the polygenic score for IQ did not significantly differentiate between these two subgroups. Collectively, these findings suggest a genetic association between cognitive decline and schizophrenia [56].

**Fig. 2:**
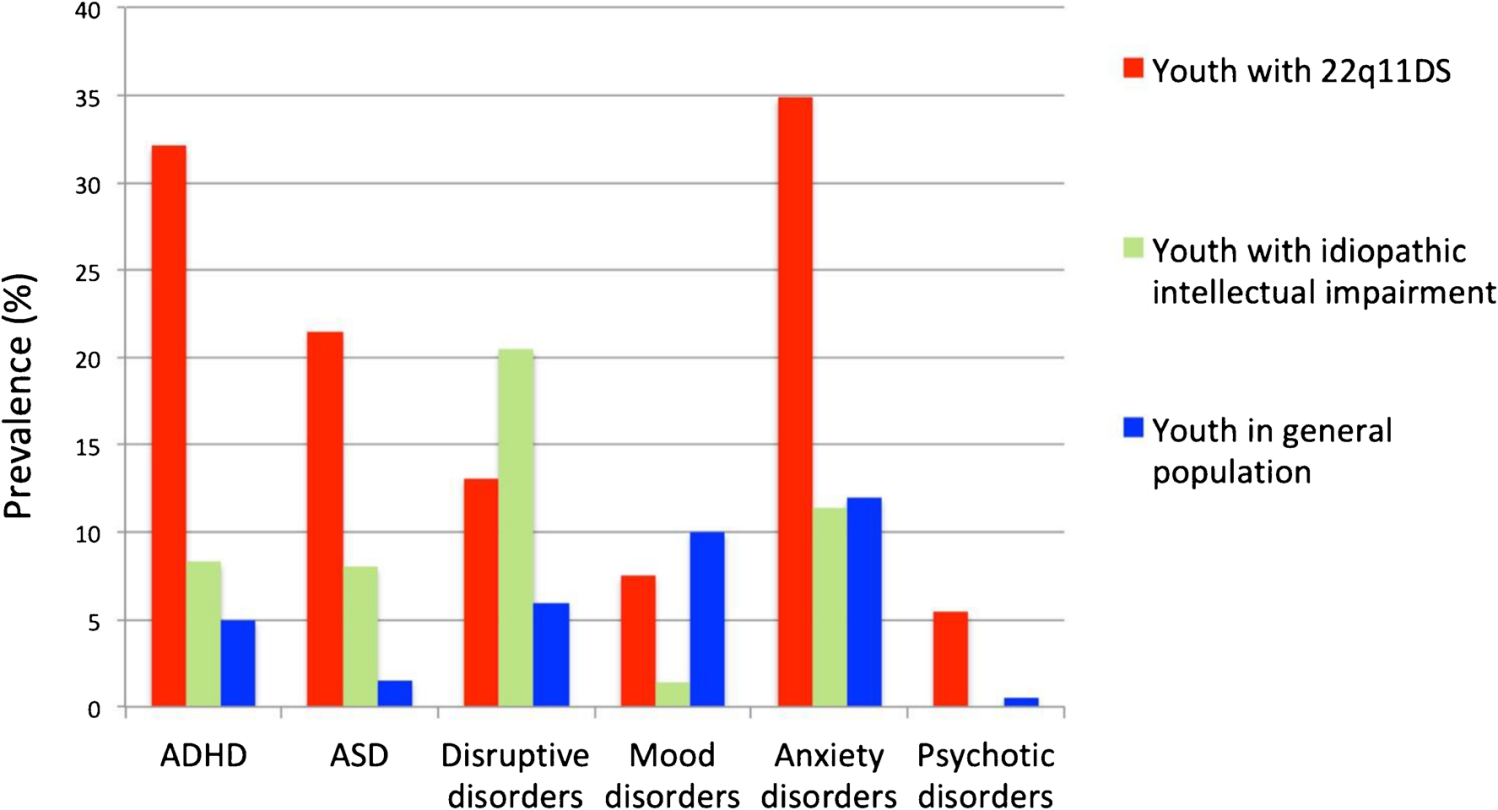
Prevalence rates of psychopathology in youth with 22q11DS compared to youth with idiopathic intellectual impairment and the general population. Figure derived from table from Fiksinski et al., Am J Med Gen A (2018). Data for youth with 22q11DS derived from Schneider et al. (2014) (805 individuals; note that the prevalence rate of psychosis is lower than the typically reported 20–25% in adults with 22q11DS, as these data only include youth). Data for youth with idiopathic intellectual impairment derived from Emerson & Hatton (2007) (641 individuals, age range 5 to 16 years, intellectual impairment based on parental/teacher report, most individuals estimated at a level of mild intellectual disability, the numbers represent point prevalence (i.e., symptoms present during the month—half year preceding the assessment); psychotic disorder not specifically reported). Data for general population youth derived from cohorts including both children and adolescents with exact age ranging vary between the different studies

At the same time, observations from epidemiological studies indicate that the overall rate of psychopathology is increased in youth with idiopathic intellectual impairment [91]. This raises the question to what extent the prevalence of neurodevelopmental and psychiatric disorders in 22q11DS deviates from what is reported in unselected cohorts with intellectual impairment. Although different methodologies hinder the direct comparison of findings between different studies, the reported profiles of psychopathology between 22q11DS and IQ-matched individuals without 22q11DS suggest that 22q11DS increases the risk of some psychiatric disorders (psychotic disorders, ASD, ADHD, anxiety, and mood disorders), but not of others (disruptive disorders, substance use disorders) (Fig. 2) [7].

Another observation in this regard is that, with the exception of psychotic disorders, studies have found no correlation between cognitive level and the risk for psychiatric disorders in individuals with 22q11DS [13, 92], although recent evidence is emerging for an age-specific association between certain specific domains of cognition and psychopathology [93]. 22q11DS thus appears to have a specific impact on the behavioral phenotype, as well as on the cognitive phenotype. This has important implications for psychiatry, highlighting that increased risk of psychopathology cannot be solely explained as an unspecific consequence of intellectual impairment [7].

## Implications for Clinical Care and Research

Clinical manifestations of 22q11DS are highly variable and can affect multiple organ systems [1]; practical guidelines have been published to assist clinicians with the management of these often complex presentations [94]. From a mental health care perspective, a focus on the genetically mediated increased risk for schizophrenia is understandable, not only because the association is strong, with approximately one in four to five individuals with 22q11DS developing this illness, but also because schizophrenia is among the most severe psychiatric conditions, with substantial impact on well-being [95] and long-term outcomes [96], including life expectancy [97]. Historically, schizophrenia was the first reported specific psychiatric phenotype in individuals with 22q11DS [4–6, 98]. Subsequent studies progressively revealed a broader scope of associated psychopathology, including anxiety and mood disorders, as well as neurodevelopmental disorders identifiable early in life, such as language disorders, intellectual disability, specific learning disorders, ADHD, and ASD.

Each of these conditions warrants appropriate diagnostic attention as well as optimal clinical guidance and treatment whenever possible. However, the psychiatric multimorbidity observed in many patients with 22q11DS carries a real danger of initiating multiple more or less independent treatment(s) based on an individual consideration of each diagnosis. Such fragmented approaches are understandable and probably at least partly also relate to how mental health care is organized. However, from the standpoint of good clinical practice, a holistic approach is likely superior, as it integrates developmental, cognitive, and behavioral aspects and includes a dimensional view on symptoms rather than relying exclusively on diagnostic categories [37]. It is likely that future developments in data-driven network approaches [99]—which strive to understand mental health issues as a set of inter-connected signs and symptoms—once applied to clinical practice, will contribute to understanding the clinical presentation of complex disorders in a more holistic approach. Importantly, such perspective decreases the risk of overshadowing, where psychopathology remains undiagnosed because relevant psychiatric symptoms are wrongly attributed to the cognitive impairment or the presence of another comorbid psychiatric condition [100, 101]. A holistic view also takes into account potential relations between the various components of the clinical presentation. For example, deficits in attention and planning may be taken to indicate the possibility of ADHD when considered independently, but when viewed in the context of comorbid developmental delay may be appropriate for mental age, given that the DSM-5 requires symptoms to be “inconsistent with developmental level” [102]. Other examples include high levels of anxiety which may sometimes be due to an uneven distribution of cognitive abilities [84] in the face of unrealistically high academic expectations; or social withdrawal, which may be understood in the context of increasing complexity of social demands gradually exceeding limited social and communicative skills. Importantly, in these examples, it is not the level of impairment in and by itself, but rather the extent to which they are out of sync with environmental demands and expectations. Indeed, studies indicate that the increased rates of psychopathology in 22q11DS are not an unspecific consequence of cognitive impairment in this population [7]. However, this may be different with regard to schizophrenia. Similar as to observations in the general population [86, 103, 104], studies in 22q11DS show that both low intellectual ability early in life and a subsequent decline thereof are associated with increased risk of schizophrenia [9]. For clinicians, these observations indicate the importance of careful monitoring of cognitive and social abilities, with attention to the (fluctuating) balance between individual abilities on the one hand, and environmental expectations on the other.

Research into 22q11DS aims to improve the quality of care for individuals and their families. However, research in this patient group has important ramifications for other clinical groups. The strong association with schizophrenia combined with the ability to make a genetic diagnosis early in life provides an unprecedented opportunity to study schizophrenia in an etiologically homogeneous subpopulation, starting early in development. This lifespan perspective is consistent not only with a neurodevelopmental perspective of schizophrenia but also for other conditions associated with 22q11DS (e.g., anxiety disorders, ADHD, and ASD). In the past 15 years or so, an increasing number of rare pathogenic genetic variants are being discovered, associated with high risks of neurodevelopmental and psychiatric outcomes [105]. Against this backdrop, 22q11DS is emerging as one of a few “least rare” among these rare genetic conditions, thereby allowing sufficiently powered studies to start examining the mechanisms underlying variable penetrance and pleiotropy, all of which are invariably observed in relation to rare genetic disorders and pose formidable challenges to both clinicians and researchers. Clinical care practices developed for 22q11DS may lead the way for clinical care of newly identified neuropsychiatric variants. As genetic testing increases within psychiatric settings, the lessons learnt from 22q11DS will become ever more vital.

## Conclusions

Since its earliest clinical descriptions of 22q11DS in the 1960s [106], a vast body of research continues to contribute to our current understanding of this complex, multisystem genetic disorder. Since the 1990s, the increased risk for schizophrenia became clear, followed by the observations of other psychopathology, including developmental disorders identifiable early in life. Subsequent and more recent studies are starting to elucidate the relations between different domains of psychopathology and the role of cognition. There is an increasing awareness to view these components in a developmental, lifespan perspective and the need to consider symptoms not only in a categorical but also in a dimensional, quantitative framework. These changing perspectives allow for a more holistic diagnostic approach and treatment for this population, but are also vital for research. Studies in the 22q11DS population continue to contribute to our understanding of schizophrenia, other developmental disorders, variable penetrance and expressivity, and pleiotropy. All of these are challenging phenomena that are relevant to neuropsychiatric outcomes in individuals with the 22q11.2 deletion as well as other rare pathogenic variants and to the understanding of the expression of, and mechanisms underlying, neuropsychiatric disorders more broadly.

### Glossary

*Pleiotropy* refers to the concept that one gene can simultaneously influence more than one distinct trait/phenotype, downstream effect, or organ. For example, cardiac malformations and schizophrenia are considered true pleiotropic manifestations in 22q11DS because they were shown to occur independently from each other [18]. Within the domain of neuropsychiatric manifestations, it is sometimes difficult to determine if two different symptoms are the expression of “true” pleiotropy or rather reflect “pseudopleiotropy” (phenotypes that are observed as separate manifestations, whereas in reality they represent the same pathological process) (for a more comprehensive discussion on this topic, see [7]).

*Incomplete, or variable, penetrance* refers to the observations that not all individuals carrying a certain genetic risk variant will display the phenotype. For example, the penetrance of schizophrenia in 22q11DS is 25%.

A closely related, but distinct concept is *variable expressivity*. Since variable expressivity refers to the observed variations in the severity of a given phenotype, it presupposes a dimensional measure of the trait. For example, the impact of the 22q11.2 deletion on intellectual ability can be expressed dimensionally as IQ; as such, it shows a pattern consistent with variable expressivity. However, when considering IQ level as a categorical trait, for example separating those with intellectual disability (ID; here defined as IQ below 70) from those without, one could say that in 22q11DS, the penetrance of ID is incomplete, with approximately 45% of individuals having an IQ in the range of ID.
